# Plastome evolution in the genus *Sium* (Apiaceae, Oenantheae) inferred from phylogenomic and comparative analyses

**DOI:** 10.1186/s12870-023-04376-8

**Published:** 2023-07-25

**Authors:** Jing Zhou, Junmei Niu, Xinyue Wang, Jiarui Yue, Shilin Zhou, Zhenwen Liu

**Affiliations:** 1 School of Pharmaceutical Science and Yunnan Key Laboratory of Pharmacology for Natural Products, Kunming Medical University, 1168 Western Chunrong Road, Yuhua Street, Chenggong New City, Kunming, China; 2 Yunnan Academy of Forestry and Grassland, Kunming, China; 3 Gaoligong Mountain, Forest Ecosystem, Observation and Research Station of Yunnan Province, Kunming, China; 4 Yunnan Key Laboratory of Biodiversity and Ecological Security of Gaoligong Mountain, Kunming, China

**Keywords:** Choloplast genome, Molecular marker, Phylogenetic relationships, *Sium*, Species identification

## Abstract

**Background:**

*Sium* L. (Apiaceae) is a small genus distributed primarily in Eurasia, with one species also occurring in North America. Recently, its circumscription has been revised to include 10 species, however, the phylogenetic relationships within its two inclusive clades were poorly supported or collapsed in previous studies based on nuclear ribosomal DNA ITS or cpDNA sequences. To identify molecular markers suitable for future intraspecific phylogeographic and population genetic studies, and to evaluate the efficacy of plastome in resolving the phylogenetic relationships of the genus, the complete chloroplast (cp) genomes of six *Sium* species were sequenced.

**Results:**

The *Sium* plastomes exhibited typical quadripartite structures of Apiaceae and most other higher plant plastid DNAs, and were relatively conserved in their size (153,029–155,006 bp), gene arrangement and content (with 114 unique genes). A total of 61–67 SSRs, along with 12 highly divergent regions (*trnQ*, *trnG-atpA*, *trnE-trnT*, *rps4-trnT*, *accD-psbI*, *rpl16*, *ycf1-ndhF*, *ndhF-rpl32*, *rpl32-trnL*, *ndhE-ndhG*, *ycf1a* and *ycf1b*) were discovered in the plastomes. No significant IR length variation was detected showing that plastome evolution was conserved within this genus. Phylogenomic analysis based on whole chloroplast genome sequences produced a highly resolved phylogenetic tree, in which the monophyly of *Sium*, as well as the sister relationship of its two inclusive clades were strongly supported.

**Conclusions:**

The plastome sequences could greatly improve phylogenetic resolution, and will provide genomic resources and potential markers useful for future studies of the genus.

**Supplementary Information:**

The online version contains supplementary material available at 10.1186/s12870-023-04376-8.

## Introduction

*Sium* L. is a small genus belonging to the tribe Oenantheae of Apiaceae subfamily Apioideae, which included 12 species in previous delimitation [1–3]. The major distribution center of the genus is Eurasia, where nine species occur (*S. ventricosum* (H.de Boissieu) L.S.Wang & M.F.Watson, *S. latifolium* L., *S. medium* Fisch. & C.A.Mey., *S. ninsi* Thunb., *S. serra* (Franch. & Sav.) Kitag., *S. sisaroideum* DC., *S. sisarum* L., *S. suave* Walter-it is also distributed in North America, and *S. tenue* Kom.). In sub-Saharan Africa, the genus has one species (*S. repandum* Welw. ex Hiern), while *Sium burchellii* (Hook. f.) Hemsl. and *S. bracteatum* (Roxb.) Cronk are endemic to the island of Saint Helena. The genus compassed species that often live in moist to aquatic habitats, and characterized by glabrous throughout, fascicled, fusiform or fibrous roots, simple pinnate leaves with sessile pinnae, long and reflexed styles, fruits with prominent and corky-thickened ribs [4].

Some species of *Sium* are used as herbal medicines in Chinese folk remedies [4]. They are rich in essential oils [5], and can be used for dispelling wind-cold, relieving headache and lowering blood pressure [6]. The thickened roots of *Sium sisarum* are rich in carbohydrates, and served as food before the potato was introduced [7, 8]. Furthermore, *Sium suave* is often sold in medicinal markets as an adulterant of “Gao-ben” [9].

The circumscription of *Sium* and its infrageneric phylogenetic relationships have been controversial. For example, members of the traditionally delimited *Berula* and *Sium* have long been considered closely related and sometimes even synonymous [10]. Moreover, the relationship between the African and the non-African members of *Sium* is unclear [1]. In recent years, several molecular phylogenetic studies have been conduted on *Sium*, and the taxonomic status of some species were resolved. Among them, *Pimpinella crispulifolia* H.de Boissieu was transferred into the *Sium* based on morphological and molecular analysis [11], and *S. serra* and *S. ventricosum* were recognized as belonging to the genus *Sium* [1]. Furthermore, the comprehensive revision of the tribe Oenantheae [12, 13], along with the molecular phylogenetic analysis of *Sium* and related genera [1, 14], showed that the genus *Sium*, as previously circumscribed (with 12 species), was shown to be polyphyletic. Its three species (*S. repandum*, *S. burchellii* and *S. bracteatum*) outside of Eurasia (from sub-Saharan African/Saint Helena), together with *Berula* (Africa, Eurasia and North America) and *Afrocarum* (Africa), formed a strongly supported clade (*Berula* s.l.). *Berula* s.l. has been recognized at the generic level and these African/Saint Helena species were thus transferred into the genus *Berula*. The remaining species constituted the *Sium* s.s., including the northern Holarctic clade (whose members generally occur in the northern Holarctic) and the southern Palearctic clade (whose members are distributed in the Palearctic, usually farther south than those of the northern Holarctic clade). However, the phylogenetic relationships between the two inclusive clades were poorly supported in the ITS trees [1, 11], or they comprised two branches of a five-way polytomy in the cpDNA (*rps16-trnK*) analysis [14]. Therefore, it is necessary to use more effective molecular markers or genomic data to investigate the phylogenetic relationship between the two clades.

Plastomes can provide valuable information for taxonomy and phylogeny, and have proven to be a powerful tool for exploring phylogenetic relationships [15, 16]. It have been applied to the comparative analysis and phylogenetic reconstruction of Apiaceae, in which the genome structure was characterized and some contentious phylogenetic relationships have been resolved [17–24]. Furthermore, highly divergent regions and simple sequence repeats (SSRs) obtained from plastome sequences can be used as efficient molecular markers for species delimitation, as well as population genetics [25].

In this study, the complete chloroplast (cp) genomes of six *Sium* species were sequenced, characterized and compared. We aimed to: (1) analyze the global features and structural patterns of *Sium* cp genomes; (2) identify simple sequence repeats (SSRs) and hotspot regions within *Sium*; (3) evaluate the efficacy of the whole cp genome in resolving the relationships within the genus, and compared with the results of ITS analysis. This is the first comprehensive analysis on cp genomes of the genus *Sium*, the results of which will provide genetic resources and molecular markers for future studies of this genus.

## Results

### Characteristics of *Sium* plastomes

**Fig. 1:**
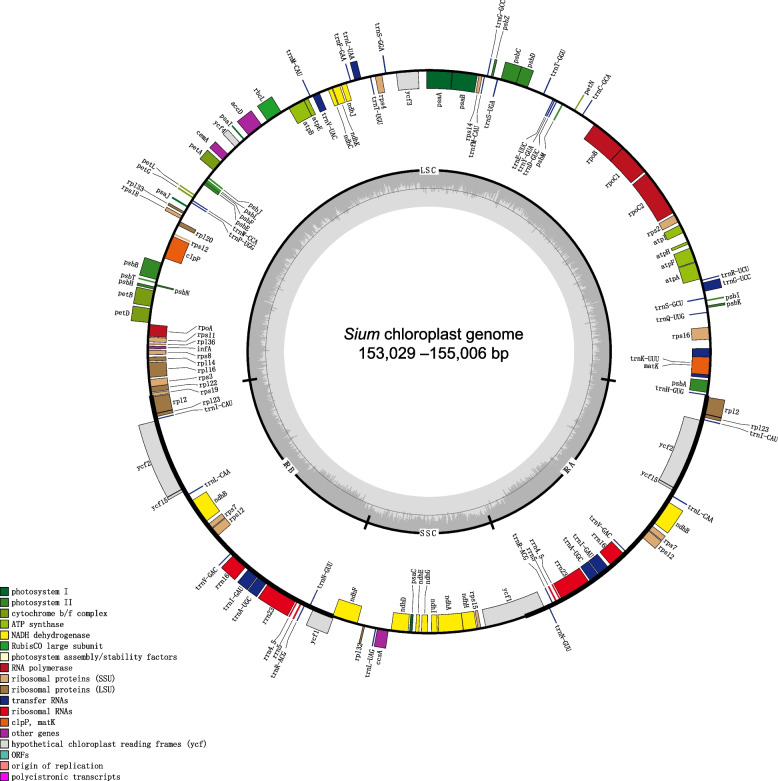
Gene map of the *Sium* plastomes. The inside and outside of the circle are genes transcribed clockwise and counterclockwise, respectively. The dashed area in the inner circle shows GC content

The seven complete cp genomes of *Sium* range in length from 153,029 bp (*S. ventricosum*) to 155,006 bp (*S. ninsi*), with the GC content identically 37.40% (Table 1). All the plastomes display a typical quadripartite structure, compose of a LSC region of 84,111–85,036 bp, a SSC region of 17,092–18,789 bp, and two inverted repeats (IRa and IRb) each of 24,970–26,472 bp (Fig. 1). They harbor 132 genes with the same arrangement order, including 87 protein-coding genes, 8 rRNA genes, and 37 tRNA genes. Most genes have a single copy, while 7 protein-coding genes (*ndhB*, *rpl2*, *rpl23*, *rps7*, *rps12*, *ycf2* and *ycf15*), 7 tRNA genes (*trnA-UGC*, *trnI-GAU*, *trnI-CAU*, *trnL-CAA*, *trnN-GUU*, *trnR-ACG* and *trnV-GAC*), and four rRNA genes (*rrn16*, *rrn23*, *rrn4.5* and *rrn5*) are duplicated in the IR regions (Fig. 1, Table S1). Of the 114 unique genes, 18 genes (including 12 protein-coding genes and 6 tRNA genes) have introns, with 16 genes (*atpF*, *ndhA*, *ndhB*, *petB*, *petD*, *rpl2*, *rpl16*, *rpoC1*, *rps12*, *rps16*, *trnA-UGC*, *trnG-UCC*, *trnI-GAU*, *trnK-UUU*, *trnL-UAA* and *trnV-UAC*) having one intron and two genes (*ycf3* and *clpP*) having two introns. The two individuals of *S. suave* present minor difference in length, with 154,642 bp (*S. suave* 1) and 154,676 bp (*S. suave* 2). In general, the plastomes of *Sium* are highly identical in their structural organization, gene order and gene content.

**Table 1:** Voucher information and complete chloroplast genome features of seven *Sium* taxa

Taxa	Nucleotide length (bp)				Number of genes			GC content				Source	Genbank no
	Total	LSC	SSC	IR	CDS	tRNA	rRNA	Total	LSC	SSC	IR		
*S. suave 1*	154,642	84,111	17,599	26,466	80	30	4	37.40%	35.50%	30.70%	42.70%	Yuanmingyuan, Beijing, China (Chen Yaodong 571, PE)	OP234519
*S. suave 2*	154,676	84,155	17,583	26,469	80	30	4	37.40%	35.50%	30.70%	42.70%	Botanical garden, Institute of Botany, CAS (S3, Kunming Medical University)	OP234520
*S. ventricosum*	153,029	84,300	18,789	24,970	80	30	4	37.40%	35.50%	30.70%	42.70%	Daocheng, Sichuan, China (LZ20160686, Kunming Medical University)	OP234514
*S. tenue*	154,929	84,952	17,569	26,204	80	30	4	37.40%	35.50%	30.70%	42.70%	Haiyang, Shandong, China (lilan668, KUN)	OP234515
*S. medium*	154,598	84,116	17,538	26,472	80	30	4	37.40%	35.50%	30.70%	42.70%	Wenquan, Xinjiang, China (SCSB-SHI-2006220, KUN)	OP234517
*S. crispulifolium*	154,099	84,707	17,092	26,150	80	30	4	37.40%	35.50%	31.00%	42.70%	Luquan, Yunnan, China ( LZ1523, Kunming Medical University)	OP234518
*S. ninsi*	155,006	85,036	17,554	26,208	80	30	4	37.40%	35.50%	30.60%	42.70%	Mikawa, Japan (Miyoshi Furuse 51,348, PE)	OP234516

### Sequence repeats analysis

**Fig. 2:**
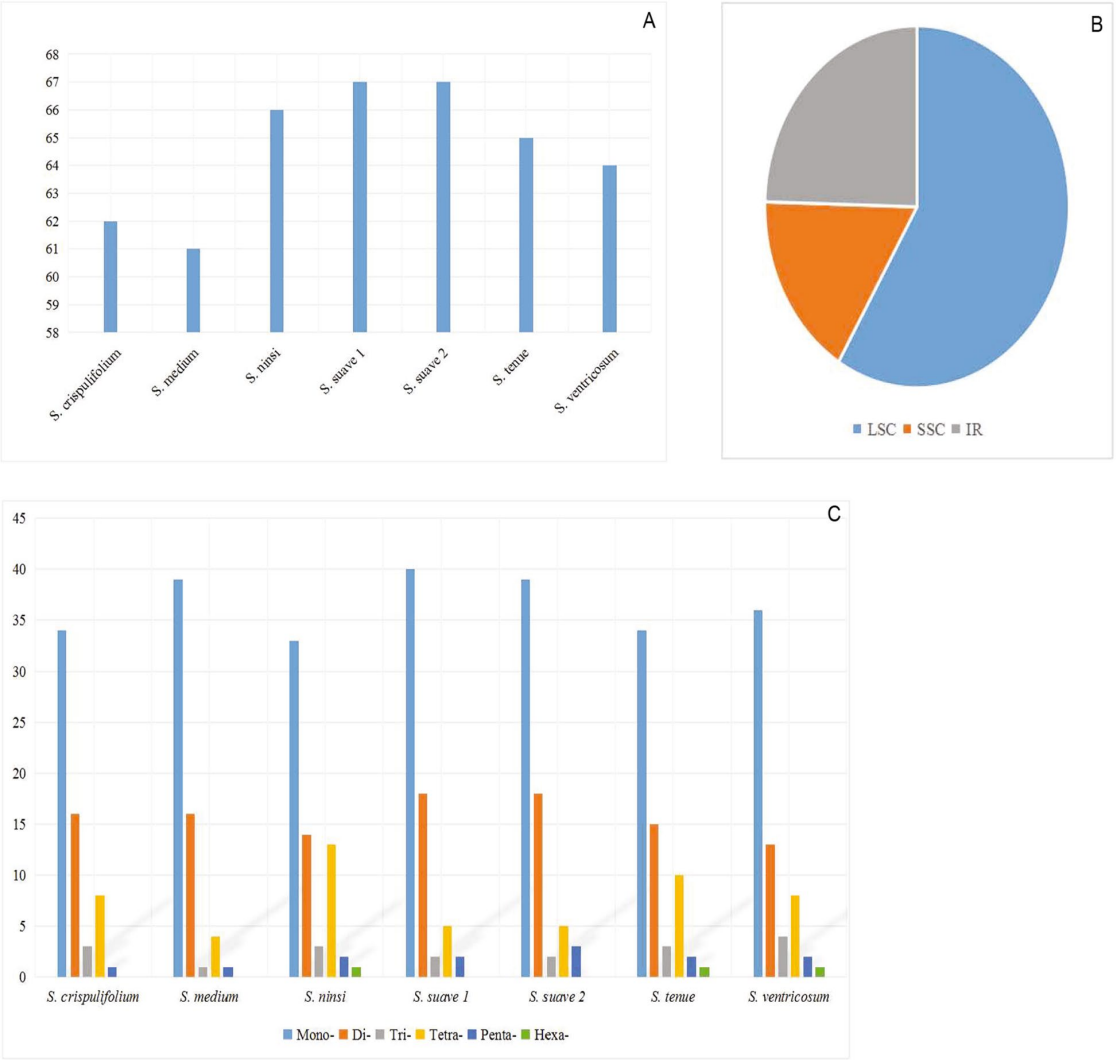
Comparison of simple sequence repeats (SSRs) among seven *Sium* chloroplast genomes. **a** Numbers of SSRs detected in chloroplast genomes; **b**. Frequencies of SSRs identified in LSC, IR and SSC regions; **c**. Types of SSRs detected in each chloroplast genomes

A total of 452 SSRs are identified in the seven *Sium* cp genomes (Fig. 2 and Table S2). The number of SSRs discovered in each species range from 61 (*S. medium*) to 67 (*S. suave*) (Fig. 2A). They are mainly located in LSC (265 SSRs, 58.63%) or IR region (111 SSRs, 24.56%), and only a minority are occurred in the SSC region (76 SSRs, 16.81%) (Fig. 2B). Among which, mono-nucleotide, di-nucleotide, tri-nucleotide, tetra-nucleotide, and penta-nucleotide repeats are detected in all species, while hexa-nucleotides SSRs (CCTATA) are only found in *S. ventricosum*, *S. tenue* and *S. ninsi* (Fig. 2C). The most abundant repeats are mono-nucleotide, which accounts for 50.00–63.93% of the total, followed by di-nucleotide repeats (20.21–26.87%), tetra-nucleotide repeats (6.56–19.70%), tri-nucleotide repeats (1.64–6.25%), and penta-nucleotide repeats have the least amount (1.61–4.48%). Meanwhile, the majority of SSRs are the A/T type (80.30–85.25%).

### Comparative genomic analysis

**Fig. 3:**
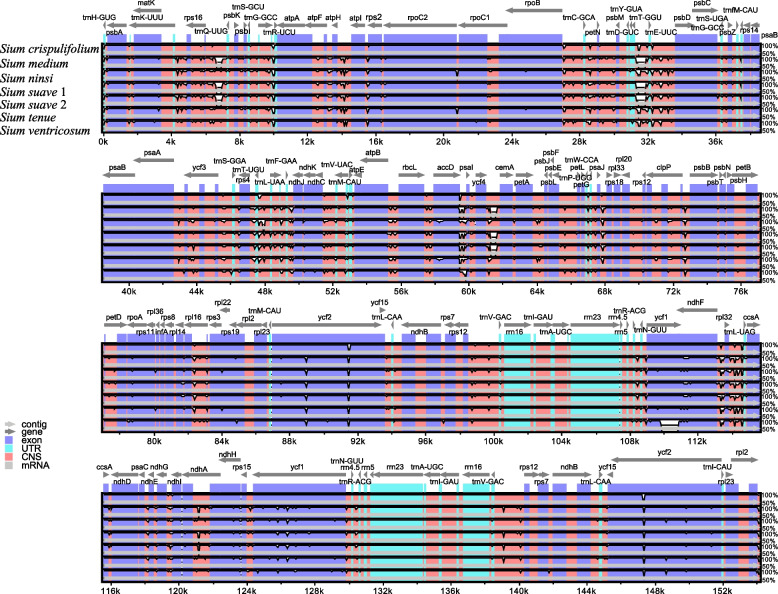
Sequence identity plots among the seven *Sium* chloroplast genomes using mVISTA, with *S. crispulifolium* as the reference

The aligned cp genomes of six *Sium* species result in a matrix of 158,319 bp, and show high sequence similarity (99.20–99.96%). In which, 2630 variable sites and 1245 parsimony informative sites are included. The divergence levels of the noncoding and single-copy regions (LSC and SSC) are higher than that of the coding and IR regions, respectively (Fig. 3). At the genome level, the sequence divergence among the six *Sium* species range from 0.0004 to 0.0080, with an average of 0.0060. Furthermore, the two species endemic to China (*S. ventricosum* and *S. crispulifolium*) show the sequence divergence of 0.0060, and the largest sequence divergence value is observed between *S. crispulifolium* and *S. medium* (0.0080).

**Fig. 4:**
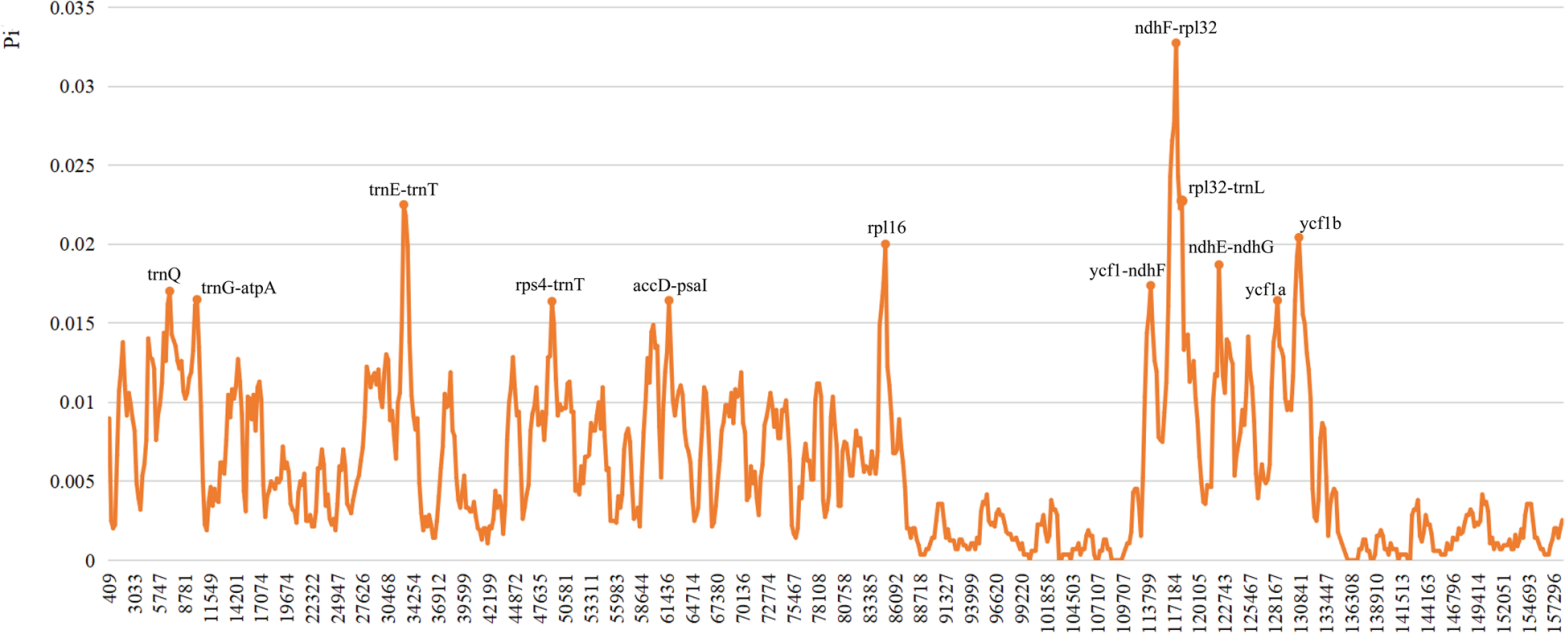
Sliding window analysis of the *Sium* chloroplast genomes (window length: 800 bp, step size: 200 bp). Mutation hotspot regions (*Pi* > 0.015) were annotated

The sliding window analyses across the seven plastomes show that the nucleotide diversity (*Pi*) range from 0.0000 to 0.0327 (Fig. 4). A total of 12 regions (*trnQ*, *trnG-atpA*, *trnE-trnT*, *rps4-trnT*, *accD-psbI*, *rpl16*, *ycf1-ndhF*, *ndhF-rpl32*, *rpl32-trnL*, *ndhE-ndhG*, *ycf1a* and *ycf1b*) are recognized as hotspot regions with nucleotide diversity > 0.015. Of those, six regions are located in the LSC, and six were in the SSC region. The NJ trees show that all these regions could identify *Sium* species separately or jointly (Figures S1 and S2). The primers are designed for the 12 variable markers (Table S3).

**Fig. 5:**
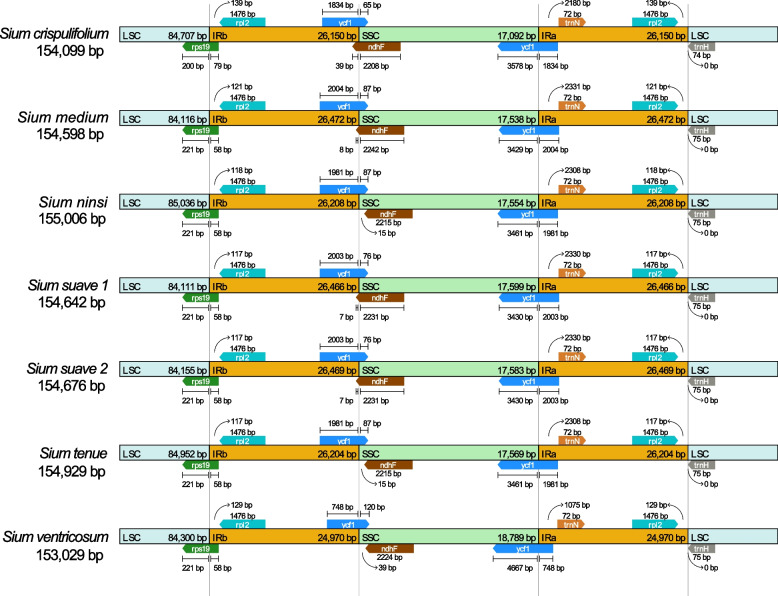
Comparisons of the borders of LSC, SSC, and IR regions among seven *Sium* chloroplast genomes

The expansion and contraction of the junction regions are analyzed for the six *Sium* species, and no significant IR length variation is detected (Fig. 5). The genes *rps19*, *rpl2*, *ycf1*, *ndhF* and *trnH* are present at the junction of the LSC/IRb, IRb/SSC, SSC/IRa and IRa/LSC borders. In all plastomes, the LSC/IRb boundary is located within the *rps19* gene, with part of it (58–79 bp) duplicated in the IRa. Similarly, the SSC/IRa junction are located in the *ycf1* gene across all species, resulting in the presence of ψ*ycf1* in the IRb. There are two copies of the *rpl2* genes located in the IR regions (IRa and IRb), which is 117–139 bp away from the LSC/IR junction, while the *trnH* gene is just located at the junction of LSC/IRa region in all plastomes. The *ndhF* gene of *S. ninsi*, *S. tenue* and *S. ventricosum* are completely located in the SSC region, which extends into the IRb region by about 7–39 bp in other species. These variations at boundary regions lead to the length variation in the cp genomes of *Sium*.

### Phylogenetic analysis

**Fig. 6:**
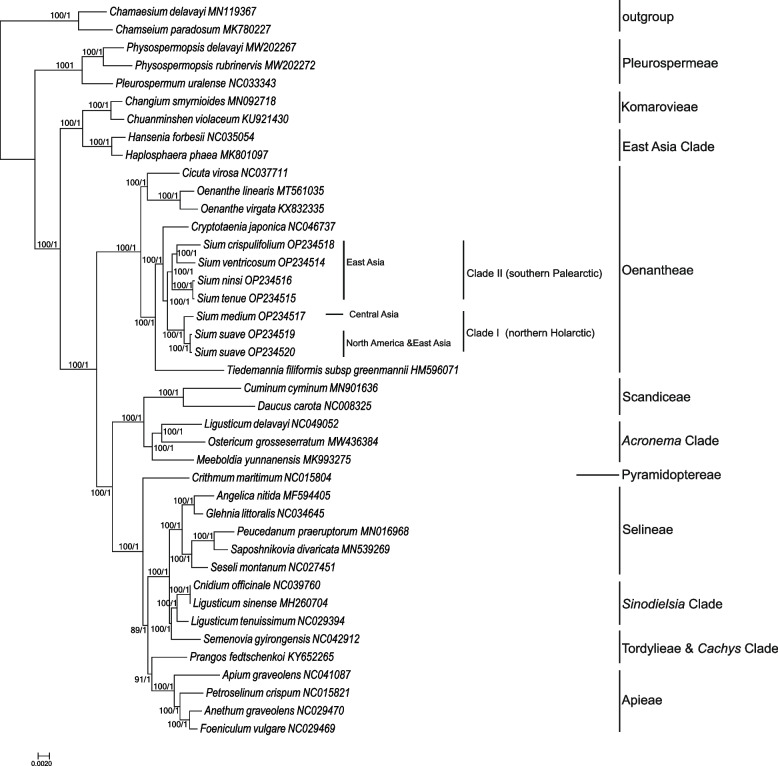
Phylogenetic relationships of 41 species inferred from Bayesian Inference (BI) and Maximum Likelihood (ML) analyses of the complete chloroplast genomes. The bootstrap support values (BS) and posterior probabilities (PP) are shown next to the branches

Using the complete cp genome sequences, phylogenetic relationships among the major clades of Apioideae, as well as six *Sium* species are inferred. The phylogenies estimated using ML and BI analyses are well-resolved and fully consistent with one another (Fig. 6). In all analyses, the monophyly of *Sium* is recovered with strong support (bootstrap value, BS = 100, PP = 1.00). Within *Sium*, two monophyletic sister clades are recognized with high support (BS = 100, PP = 1.00). Clade I includes two species distributed in the northern Holarctic [1, 14]: central Asiatic *S. medium* is sister group to the clade of eastern Asiatic-North American *S. suave*. The remaining species of the genus constitute the Clade II, in which the two species endemic to China (*S. ventricosum* and *S. crispulifolium*) ally together, and comprise a sister group relationship to the group of *S. ninsi* and *S. tenue*. The ITS and plastome trees produce consistent topologies for *Sium* species (Figure S3).

## Discussion

In this study, we assembled and annotated the complete plastid genome sequences of six *Sium* species for the first time. Having a range of 153,029–155,006 bp, the genome sizes of *Sium* fell within the typical size range of Apiaceae plastomes [17–25]. Furthermore, all plastomes are typical of Apiaceae and most other angiosperms plastid DNAs in terms of structural organization, gene arrangement and content. The cp genomes of *Sium* species had an average GC content of 37.40%, similar to those previously published Apiaceae genomes [22, 23]. Moreover, the GC contents in LSC and SSC regions were significantly lower than those in the IR region, as the rRNA genes with high GC content located in IR regions. In Apiaceae, the expansion and contraction of the IR region, and the introgression of mitochondrial DNA into the plastid genome have been previously reported for some species [25–30]. In the present study, however, no significant IR length variation was detected among *Sium* plastomes, showing that plastome evolution was conserved within this genus.

To better resolve relationships among closely related species, great efforts have been made to identify more variable chloroplast regions [31–33]. Across Apiaceae lineages, the *rpl32-trnL*, *trnE-trnT*, *ndhF-rpl32*, *rps16-trnQ* and *trnT-psbD* intergenic spacers are among the most fast-evolving loci, while the *trnD-trnY-trnE-trnT* combined region presents the greatest number of potentially parsimony informative characters [25]. In which, *rps16-trnQ*, *rpl32-trnL* intergenic spacers and *rps16* introns have previously been considered useful in resolving low-level relationships of Apiaceae [34–38]. In addition to these loci, we found that a total of nine regions held a relatively higher *Pi* values for *Sium*: *trnQ*, *trnG-atpA*, *rps4-trnT*, *accD-psbI*, *rpl16*, *ycf1-ndhF*, *ndhE-ndhG*, *ycf1a* and *ycf1b*. Although some regions yielded species relationships that differed from the genomic results, all *Sium* species could be successfully identified on the neighbor-joining trees. Therefore, these regions can be used as lineage-specific DNA barcodes in future plant identification and speciation studies in *Sium*. SSRs play an important role as molecular markers in plant population genetics, evolutionary and ecological studies due to their high levels of mutation rates and polymorphism [39]. In this study, we identified 61 to 67 SSRs in seven *Sium* plastomes, which will be conducive to the assessment of genetic differentiation within and among populations [30].

In recent years, several molecular studies on tribe Oenantheae and the genus *Sium*, based on nuclear rDNA ITS and few cpDNA sequences, have been carried out [1, 12–14]. In all of these studies, *Sium* is resolved as a polyphyletic group, as its African and Saint Helena members (*S. repandum*, *S. bracteatum* and *S. burchellii*), along with the monotypic African *Afrocarum*, were nested within *Berula* forming the *Berula* s.l. clade. These four African/Saint Helena species have been transferred into the genus *Berula* [14]. The remaining nine species of *Sium* constituted the *Sium* s.s., and consisted of two clades: the northern Holarctic clade (*S. latifolium*, *S. medium* and *S. suave*) and the southern Palearctic clade (included *S. ventricosum*, *S. ninsi*, *S. serra*, *S*. *sisaroideum*, *S. sisarum* and *S. tenue*). In all analyses, the monophyly of each of the two clades is strongly supported, however, their sister group relationship was poorly supported or collapsed in previous studies [1, 11, 14]. With the use of complete cp genome sequences, a highly consistent topology was recovered, the monophyly of the northern Holarctic clade and the southern Palearctic clade, and their sister relationship were strongly supported. *Sium crispulifolium* was a poorly known species endemic to China, and it was initially described under *Pimpinella* as *P. crispulifolia* H.de Boissieu [40]. However, this placement was not certain because the type specimen has no ripe fruits. Recently, based on molecular affinity, and morphological similarity to *Sium* (e.g., fascicled root, long and reflexed styles, conspicuous calyx teeth, and fruits with corky-thickened ribs), it was transferred into the *Sium* [11]. Thus, the circumscription of *Sium* was expanded to accommodate 10 species. *Sium ventricosum* is an aquatic and dwarf plant endemic to high montane regions in southwest of China [4], which has been variously treated as species of *Apium*, *Chamaesium*, *Sium*, and *Sinocarum* [41–43]. In the present analysis, *Sium ventricosum* and *S. crispulifolium* constituted a well-supported sister group, and allied strongly with two other eastern Asia representatives (*S. tenue* and *S. ninsi*) within Clade II. Therefore, phylogenomic analysis further confirmed that they belong to the genus *Sium*. The phylogenetic positions of other *Sium* species were generally consistent with those inferred by previous studies [1, 11, 14], but with higher support.

## Conclusions

In this study, the cp genomes of six *Sium* species were analyzed and compared. The results revealed that the *Sium* plastomes are conserved in structural organization, gene order and content. The SSRs and hotspot regions identified can be used as molecular markers in the future intraspecific diversity study of *Sium*. Furthermore, our study showed that plastome sequences could greatly improve phylogenetic resolution. Overall, the complete plastome sequences reported herein enriched the genomic information available for *Sium*.

## Methods

### Taxon sampling and genome sequencing

A total of seven accessions, representing six *Sium* species were sampled for analysis (Table 1). The collection and voucher information are provided in Table 1. All samples were initially identified by the first author, then their respective ITS sequences were extracted from sequenced genomes and compared with that of Spalik et al. [14] for confirmation (the accession number for comparison are as follows: *S. ninsi*, DQ005678; *S. suave*, DQ005695; *S. ventricosum*, DQ005665; *S. tenue*, DQ005706; *S. medium*, DQ005674). DNA extractions were conducted using the Plant Genomic DNA Kit from Tiangen Biotech (Beijing) Co., Ltd., China. According to the manufacturer’s instructions, the total DNA was fragmented ultrasonically and used for 350-bp insert libraries construction. All of the genomic data were sequenced using the Illumina platform at Novogene (Beijing, China), with the paired-end reads 2 × 150 bp.

### Assembling and annotation

Raw sequence reads were quality trimmed using Trimmomatic v.0.36 [44]. Remaining high-quality reads were assembled de novo into contigs in SPAdes v3.6.1 [45]. and GetOrganelle v1.6.4 [46] with default settings. Contigs were connected with the help of Bandage version 0.8.1 [47], and manually checked when necessary. The cp genomes were annotated using CpGAVAS2 [48], and further manually adjusted in Geneious v.9.0.5 [49] according to comparisons with the plastome of *Tiedemannia filiformis* subsp. *greenmannii* (Mathias & Constance) M.A. Feist & S.R. Downie [25]. The tRNA genes were confirmed with tRNA scan-SE [50]. The circular plastid genome maps were drawn using the Organellar GenomeDRAW [51]. The seven *Sium* cp genomes and ITS sequences were submitted to GenBank (accession numbers for plastomes and ITS are OP234514–OP234520 and OR116133-OR116139, respectively).

### Simple repeat sequence analysis

The simple sequence repeats (SSRs) of *Sium* plastomes were identified with MISA [52]. Thresholds for the minimum number of repeats were 10, 5, 4, 3, 3, and 3 for mono-, di-, tri-, tetra-, penta-, and hexa nucleotides, respectively.

### Genome comparison and analysis

The mVISTA program was used to evaluate the variability of the seven complete cp genome sequences under Shuffle-LAGAN mode [53], using *S. crispulifolium* as a reference. To determine the nucleotide diversity (*Pi*) among the cp genomes of *Sium*, the sliding window analysis was conducted using DnaSP v.6.10 [54], with a step size of 200 bp and a window length of 800 bp. The primers for amplifying the highly variable regions were designed using Primer Premier 6 software (Premier, Vancouver, Canada). We also compared junction sites of LSC-IRa/b and SSC-IRa/b with IRscope [55] to detect IR expansion or contraction in the genomes of the *Sium* species.

### Phylogenomic analysis

In total, forty-one cp genomes, representing the taxa from major clades of Apioideae and seven newly obtained taxa, were used in the phylogenetic analysis. Two *Chamaesium* taxa were selected as outgroup because the genus holds a sister-group relationship to all other apioid genera, excluding those of its most early-diverging branches [56, 57]. Fourthermore, the ITS sequences of the above taxa were analyzed for comparison with the genomic results. All sequences were aligned using the MAFFT v 7.017 [58], with the Q-INS-I algorithm [59], and manually adjusted where necessary using the BioEdit sequence alignment editor [60]. Maximum likelihood (ML) analysis was conducted using RAxML version 8.1.11 [61] using the GTR + G substitution model, with 1000 bootstrap replicates; other parameters were used as the default settings. Bayesian inference (BI) analysis was carried out with MrBayes version 3.2.3 [62]. Four Markov chains starting with a random tree were run simultaneously for 1,000,000 generations, sampling trees at every 1000th generation. Convergence was assessed by examining the average standard deviation of split frequencies (ASDF) < 0.01. Trees from the first 25% were discarded as burn-in, and the remaining trees were used to build the fifty-percent majority-rule consensus tree and calculate the posterior probability (PP).

To verify the ability of highly divergent regions to identify *Sium* species, we performed phylogenetic analysis on these regions separately or jointly using MEGA 7.0. Neighbor-Joining (NJ) trees were calculated according to the Kimura 2-parameter (K2P) model using a bootstrap test with 1000 replicates.

## Supplementary Information

**Additional file 1: Figure S1.** Neighbor-Joining trees constructed for the 12 variable regions. A. *ndhE-ndhG*; B. *ndhF-rpl32*; C. *rpl16*; D. *rpl32-trnL*; E. *rps4-trnT*; F. *trnE-trnT*; G. *trnG-atpA*; H. *trnQ*; I. *ycf1a*; J: *ycf1b*; K. *ycf1-ndhF*; L. *accD-psbI.*

**Additional file 2: Figure S2.** Neighbor-Joining tree constructed for the combined 12 variable regions.

**Additional file 3: Figure S3.** Phylogenetic relationships of *Sium* species inferred from Bayesian Inference (BI) and Maximum Likelihood (ML) analyses of the ITS sequences. Bootstrap values (BS) and posterior probabilities (PP) are shown next to the branches. – indicated that the branch has no support value from BI analysis.

**Additional file 4: Table S1.** List of genes found in the *Sium* chloroplast genome.

**Additional file 5: Table S2.** More detail of SSRs in *Sium* species.

**Additional file 6: Table S3.** Primers for PCR amplification of the 12 variable regions among the seven *Sium* taxa.

## Data Availability

The seven plastomes generated in this study are available in GenBank of the National Center for Biotechnology Information (NCBI) (https://www.ncbi.nlm.nih.gov; accession numbers are OP234514-OP234520; see Table 1); Voucher specimens are deposited in KUN and PE,and collection information was listed in Table 1. Raw sequence reads can be accessed by SRR21700072 for *Sium crispulifolium*_LZ1523, SRR21700479 for *Sium medium*_SCSB-SHI-2006220, SRR21707307 for *Sium ninsi*_Miyoshi Furuse 51,348, SRR21707790 for *Sium suave*_Chen Yaodong 571, SRR21721062 for *Sium suave*_S3, SRR21721663 for *Sium tenue*_lilan668, and SRR21732700 for *Sium ventricosum*_LZ20160686.

## Abbreviations

ASDF: Average standard deviation of split frequencies
BI: Bayesian inference
BS: Bootstrap value
Cp: Complete chloroplast
IRs: Inverted regions
LSC: Large single copy region
ML: Maximum likelihood
Pi: Nucleotide diversity
PP: Posterior probability
SSC: Small single copy region
SSRs: Simple sequence repeats

## References

[1] Spalik K, Downie SR (2006). The evolutionary history of *Sium* sensu lato (Apiaceae): dispersal, vicariance, and domestication as inferred from ITS rDNA phylogeny. Amer J Bot..

[2] Mukherjee PK, Constance L (1991). Umbelliferae (Apiaceae) of India.

[3] Pimenov MG, Leonov MV (1993). *The Genera of the Umbelliferae: A Nomenclator*.

[4] Sheh ML, Watson MF. Apiaceae. In: Wu ZY, Raven PH, editors. Flora of China, vol. 14 Beijing: Science Press; St. Louis: Missouri Botanical Garden Press; 2005. p. 1–205.

[5] Öztürk G, Demirci B, Duran A, Altinordu F, Başer KHC (2017). Chemical composition and antioxidant activity of *Sium sisarum* essential oils. Nat Volatiles & Essent Oils.

[6] Zhang HK (1994). Summary of Chinese traditional medicine resources.

[7] Rostafiński J (1884). *Kucmerka (Sium sisarum) pod wzgledem geograficzno-botanicznym i historyi kultury*.

[8] Harvey JH (1984). Vegetables in the middle ages. Gard Hist.

[9] Liu ZW, Gao YZ, Zhou J (2019). Molecular authentication of the medicinal species of *Ligusticum* (Ligustici Rhizoma et Radix, “Gao-ben”) by integrating non-coding internal transcribed spacer2 (ITS2) and its secondary structure. Front Plant Sci.

[10] Drude CGO. Umbelliferae. In: Engler A, Prantl, K (eds.), Die natürlichen Pflanzenfamilien, vol. 3. Engelmann, Leipzig; 1897–1898a. p. 63–250.

[11] Zhou J, Niu JM, Guo MJ, Wang P, Liu ZW. Rediscovery of *Pimpinella crispulifolia* (Apiaceae) after one century and its new phylogenetic placement in *Sium* Phytotaxa. 2020;487: 157–163. 10.11646/phytotaxa.487.2.6.

[12] Hardway TM, Spalik K, Watson MF, Katz-Downie DS, Downie SR (2014). Circumscription of Apiaceae tribe Oenantheae. S African J Bot..

[13] Downie SR, Katz-Downie DS, Sun FJ, Lee CS (2008). Phylogeny and biogeography of Apiaceae tribe Oenantheae inferred from nuclear rDNA ITS and cpDNA psbI-5′trnK (UUU) sequences, with emphasis on the North American endemics clade. Botany.

[14] Spalik K, Downie SR, Watson MF (2009). Generic delimitations within the Sium alliance (Apiaceae tribe Oenantheae) inferred from cpDNA rps16–5′trnK (UUU) and nrDNA ITS sequences. Taxon.

[15] Zhang X, Deng T, Moore MJ, Ji YH, Lin N, Zhang HJ, Meng AP, Wang HC, Sun YX, Sun H (2019). Plastome phylogenomics of Saussurea (Asteraceae: Cardueae). BMC Plant Biol.

[16] Feng SG, Zheng KX, Jiao KL, Cai YC, Chen CL, Mao YY, Wang LY, Zhan XR, Ying QC, Wang HZ (2020). Complete chloroplast genomes of four *Physalis* species (Solanaceae): lights into genome structure, comparative analysis, and phylogenetic relationships. BMC Plant Biol.

[17] Ruhlman T, Lee SB, Jansen RK, Hostetler JB, Tallon LJ, Town CD, Daniell H (2006). Complete plastid genome sequence of Daucus carota: Implications for biotechnology and phylogeny of angiosperms. BMC Genomics.

[18] Spooner DM, Ruess H, Iorizzo M, Senalik D, Simon P (2017). Entire plastid phylogeny of the carrot genus (*Daucus*, Apiaceae): Concordance with nuclear data and mitochondrial and nuclear DNA insertions to the plastid. Amer J Bot..

[19] Yang J, Yue M, Niu C, Ma XF, Li ZH (2017). Comparative analysis of the complete chloroplast genome of four endangered herbals of Notopterygium. Genes.

[20] Kang L, Xie D, Xiao Q, Peng C, Yu Y, He XJ (2019). Peer J..

[21] Li WJ, Su ZH, Yang L, Cao QM, Feng Y (2020). Genetic diversity of the critically endangered Ferula sinkiangensis K.M. Shen (Apiaceae) and the implications for conservation. Turk J Bot.

[22] Wang ML, Wang X, Sun JH, Wang YH, Ge Y, Dong WP, Yang QJ, Huang LQ (2021). Phylogenomic and evolutionary dynamics of inverted repeats across *Angelica* plastomes. BMC Plant Biol.

[23] Wen J, Xie DF, Price M, Ren T, Deng YQ, Gui LJ, He XJ (2021). Backbone phylogeny and evolution of Apioideae (Apiaceae): New insights from phylogenomic analyses of plastome data. Mol Phylogenet Evol..

[24] Weng L, Jiang YH, Wang Y, Zhang XM, Zhou P, Wu M, Li HZ, Sun H, Chen ST (2023). Chloroplast genome characteristics and phylogeny of the sinodielsia clade (apiaceae: apioideae). BMC Plant Biol.

[25] Downie SR, Jansen RK (2015). A comparative analysis of whole plastid genomes from the Apiales: Expansion and contraction of the inverted repeat, mitochondrial to plastid transfer of DNA, and identification of highly divergent noncoding regions. Syst Bot.

[26] Goremykin VV, Salamini F, Velasco F, Viola R (2009). Mitochondrial DNA of Vitis vinifera and the issue of rampant horizontal gene transfer. Mol Biol Evol..

[27] Iorizzo M, Grzebelus D, Senalik D, Szklarczyk M, Spooner D, Simon P (2012). Against the traffic: The first evidence for mitochondrial DNA transfer into the plastid genome. Mobile Genetic Elements.

[28] Iorizzo M, Senalik D, Szklarczyk M, Grzebelus D, Spooner D, Simon P (2012). De novo assembly of the carrot mitochondrial genome using next generation sequencing of whole genomic DNA provides first evidence of DNA transfer into an angiosperm plastid genome. BMC Plant Biol.

[29] Peery R, Downie SR, Jansen RK, Raubeson LA. Apiaceae organellar genomes. In *Proceedings of the of the 18th International Botanical Congress*, Melbourne, Australia. Ruggell: A.R.G. Ganter Verlag. 2015. p. 481.

[30] Mustafina FU, Yi DK, Choi K, Shin CH, Tojibaev KS, Downie SR (2018). A comparative analysis of complete plastid genomes from Prangos fedtschenkoi and Prangos lipskyi (Apiaceae). Ecol Evol..

[31] Shaw J, Lickey EB, Beck JT, Farmer SB, Liu W, Miller J, Siripun KC, Winder CT, Schilling EE, Small RL (2005). The tortoise and the hare II: relative utility of 21 noncoding chloroplast DNA sequences for phylogenetic analysis. Amer J Bot..

[32] Shaw J, Lickey EB, Shilling EE, Small RL (2007). Comparison of whole chloroplast genome sequences to choose noncoding regions for phylogenetic studies in angiosperms: The tortoise and the hare III. Amer J Bot..

[33] Shaw J, Shafer HL, Leonard OR, Kovach MJ, Schorr M, Morris AB (2014). Chloroplast DNA sequence utility for the lowest phylogenetic and phylogeographic inferences in angiosperms: The tortoise and the hare IV. Amer J Bot..

[34] Calviño CI, Martínezc SG, Downie SR (2008). The evolutionary history of Eryngium (Apiaceae, Saniculoideae): Rapid radiations, long distance dispersals, and hybridizations. Mol Phylogenet Evol..

[35] Zhou J, Gong X, Downie SR, Peng H (2009). Towards a more robust molecular phylogeny of Chinese Apiaceae subfamily Apioideae: additional evidence from nrDNA ITS and cpDNA intron (rpl16 and rps16) sequences. Mol Phylogenet Evol.

[36] Liao CY, Downie SR, Yu Y, He XJ (2012). Historical biogeography of the Angelica group (Apiaceae tribe Selineae) inferred from analyses of nrDNA and cpDNA sequences. J Syst Evol..

[37] Fernández M, Ezcurra C, Calviño CI (2017). Chloroplast and ITS phylogenies to understand the evolutionary history of southern South American *Azorella*, *Laretia* and *Mulinum* (Azorelloideae, Apiaceae). Mol Phylogenet Evol.

[38] Ottenlips MV, Feist MAE, Mansfield D, Plunkett GM, Buerki S, Smith JF (2020). Int J Plant Sci..

[39] Govindaraj M, Vetriventhan M, Srinivasan M (2015). Importance of genetic diversity assessment in crop plants and its recent advances: an overview of its analytical perspectives. Genet Res Int..

[40] Boissieu H (1909). Note complementaire sur quelques Ombelliferes nouvelles ou peu connues d’Extreme-Orient, d’apres les collections du Museum d’Histoire naturelle de Paris. Bull Soc Bot France..

[41] Handel-Mazzetti HRE (1933). Symbolae Sinicae, Botanische Ergebnisse der Expedition der Akademie der Wissenschaften in Wien nach Sudwest-China. 1914/1918 (Umbelliferae).

[42] Pu FD, Wang WT, Wu SG, Lang KY, Li PQ, Pu FD, Chen SK (1993). Umbelliferae. Vascular Plants of Hengduan Mountains.

[43] Pimenov MG, Kljuykov EV (2003). What is Sium frigidum Hand.-Mazz. (Umbelliferae)?. Feddes Repert.

[44] Bolger AM, Lohse M, Usadel B (2014). Trimmomatic: a flexible trimmer for Illumina sequence data. Bioinformatics.

[45] Bankevich A, Nurk S, Antipov D, Gurevich AA, Dvorkin M, Kulikov AS, Lesin VM, Nikolenko SI, Pham S, Prjibelski AD, Pyshkin AV, Sirotkin AV, Vyahhi N, Tesler G, Alekseyev MA, Pevzner PA (2012). SPAdes: a new genome assembly algorithm and its applications to single-cell sequencing. J Comput Biol..

[46] Jin JJ, Yu WB, Yang JB, Song Y, dePamphilis CW, Yi TS, Li DZ (2020). GetOrganelle: a fast and versatile toolkit for accurate de novo assembly of organelle genomes. Genome Biol.

[47] Wick RR, Schultz MB, Zobel J, Holt KE (2015). Bandage: interactive visualization of de novo genome assemblies. Bioinformatics.

[48] Shi L, Chen H, Jiang M, Wang L, Wu X, Huang L, Liu C (2019). CPGAVAS2, an integrated plastome sequence annotator and analyzer. Nucleic Acids Res.

[49] Kearse M, Moir R, Wilson A, Stones-Havas S, Cheung M, Sturrock S, Buxton S, Cooper A, Markowitz S, Duran C, Thierer T, Ashton B, Meintjes P, Drummond A (2012). Geneious basic: an integrated and extendable desktop software platform for the organization and analysis of sequence data. Bioinformatics.

[50] Lowe TM, Eddy SR (1997). tRNAscan-SE: a program for improved detection of transfer RNA genes in genomic sequence. Nucleic Acids Res.

[51] Lohse M, Drechsel O, Kahlau S, Bock R (2013). Organellargenomedraw–a suite of tools for generating physical maps of plastid and mitochondrial genomes and visualizing expression data sets. Nucleic Acids Res.

[52] Beier S, Thiel T, Munch T, Scholz U, Mascher M (2017). MISA-web: a web server for microsatellite prediction. Bioinformatics.

[53] Frazer KA, Pachter L, Poliakov A, Rubin EM, Dubchak I (2004). VISTA: computational tools for comparative genomics. Nucleic Acids Res.

[54] Rozas J, Ferrer-Mata A, Sanchez-DelBarrio JC, Guirao-Rico S, Librado P, Ramos-Onsins SE, Sánchez-Gracia A (2017). DnaSP 6: DNA sequence polymorphism analysis of large data sets. Mol Biol Evol..

[55] Amiryousefi A, Hyvonen J, Poczai P (2018). IRscope: an online program to visualize the junction sites of chloroplast genomes. Bioinformatics.

[56] Downie SR, Plunkett GM, Watson MF, Spalik K, Katz-Downie DS, Valiejo-Roman CM, Terentieva EI, Troitsky AV, Lee BY, Lahham J, El-Oqlah A (2001). Tribes and clades within Apiaceae subfamily Apioideae: the contribution of molecular data. Edinb J Bot.

[57] Calviño CI, Tilney PM, Van Wyk BE, Downie SR (2006). A molecular phylogenetic study of southern African Apiaceae. Am J Bot.

[58] Katoh K, Standley DM (2013). MAFFT multiple sequence alignment software version 7: improvements in performance and usability. Mol Biol Evol..

[59] Katoh K, Toh H (2008). Recent developments in the MAFFT multiple sequence alignment program. Brief Bioinform..

[60] Hall T. BioEdit: biological sequence alignment editor for Win95/98/ NT/2K/ XP version 6.0.7. 1999; http://www.mbio.ncsu.edu/BioEdit/bioedit.html.

[61] Stamatakis A (2014). RAxML version 8: a tool for phylogenetic analysis and post-analysis of large phylogenies. Bioinformatics.

[62] Ronquist F, Huelsenbeck JP (2012). MrBayes 3 2: efficient Bayesian phylogenetic inference and model choice across a large model space. Syst Biol..

