# Supplementary figures and images for: Plastome evolution in the genus Sium (Apiaceae, Oenantheae) inferred from phylogenomic and comparative analyses
Source: BMC Plant Biol. 2023 Jul 25;23:368. doi: 10.1186/s12870-023-04376-8 (PMC10367252; doi:10.1186/s12870-023-04376-8)

A

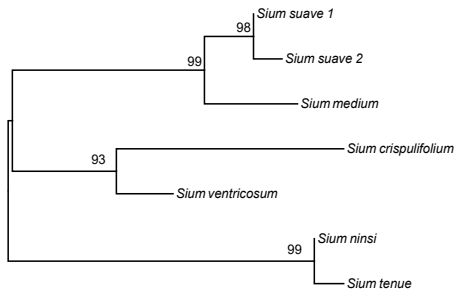

B

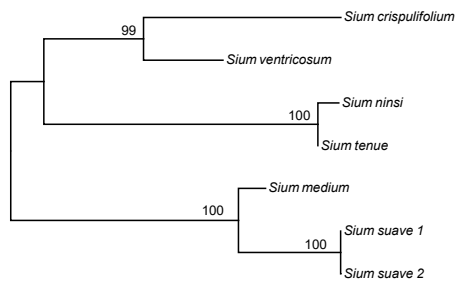

C

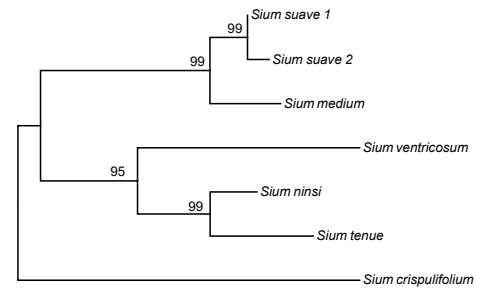

D

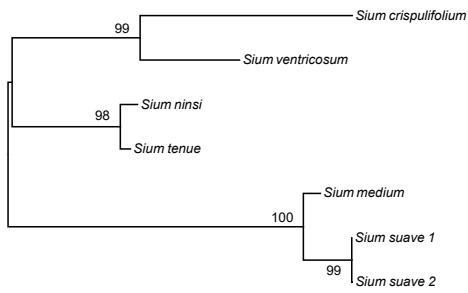

E

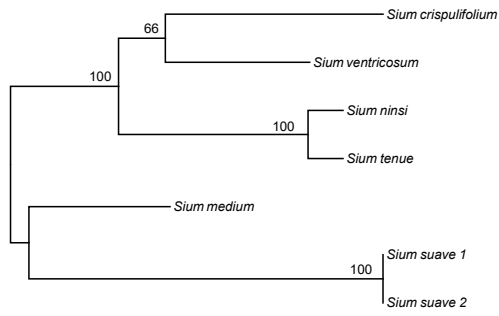

F

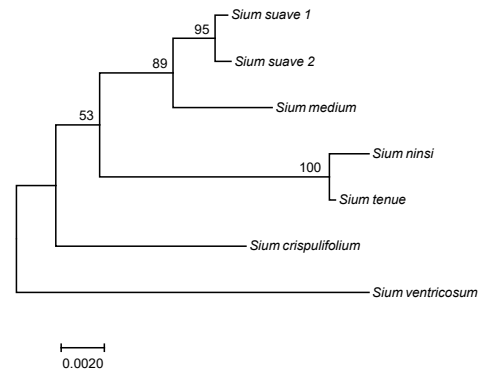

G

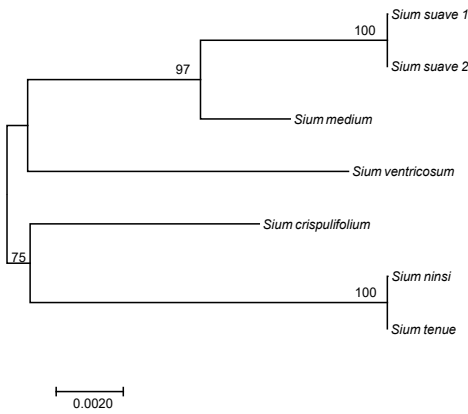

H

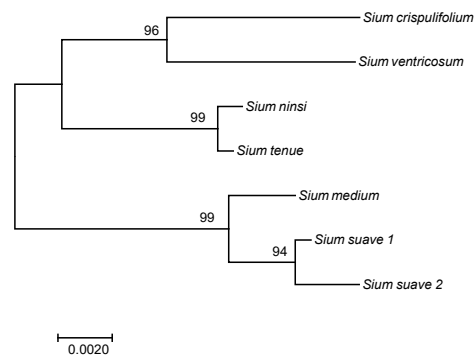

I

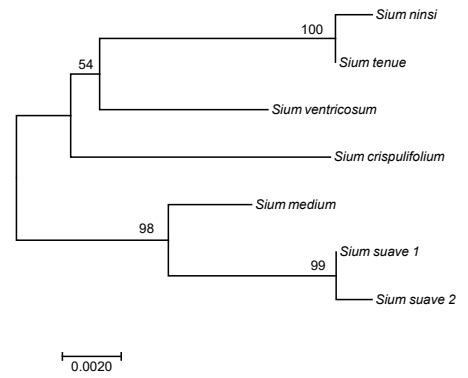

J

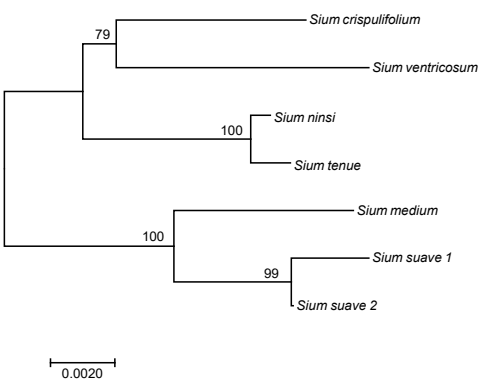

K

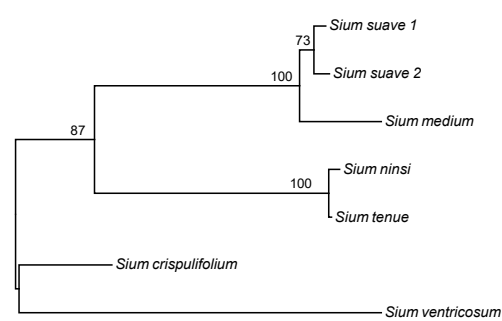

L

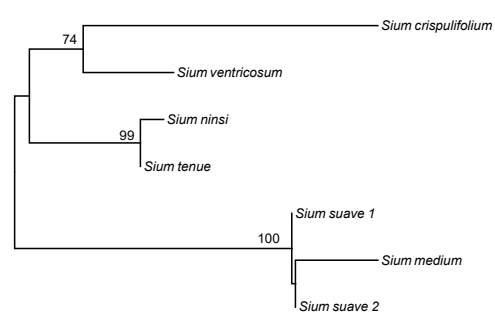

Supplement: Supplementary file 1 — Additional file 1: Figure S1. Neighbor-Joining trees constructed for the 12 variable regions. A. ndhE-ndhG; B. ndhF-rpl32; C. rpl16; D. rpl32-trnL; E. rps4-trnT; F. trnE-trnT; G. trnG-atpA; H. trnQ; I. ycf1a; J: ycf1b; K. ycf1-ndhF; L. accD-psbI. [file 12870_2023_4376_MOESM1_ESM.pdf]

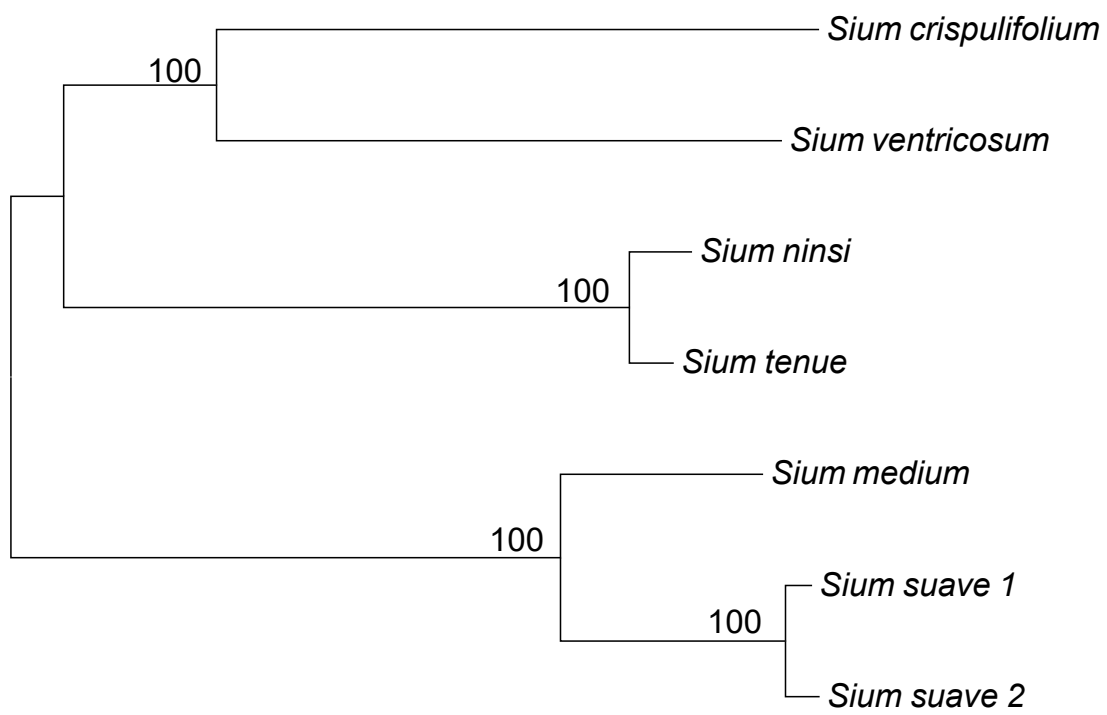

0.0020

Supplement: Supplementary file 2 — Additional file 2: Figure S2. Neighbor-Joining tree constructed for the combined 12 variable regions. [file 12870_2023_4376_MOESM2_ESM.pdf]

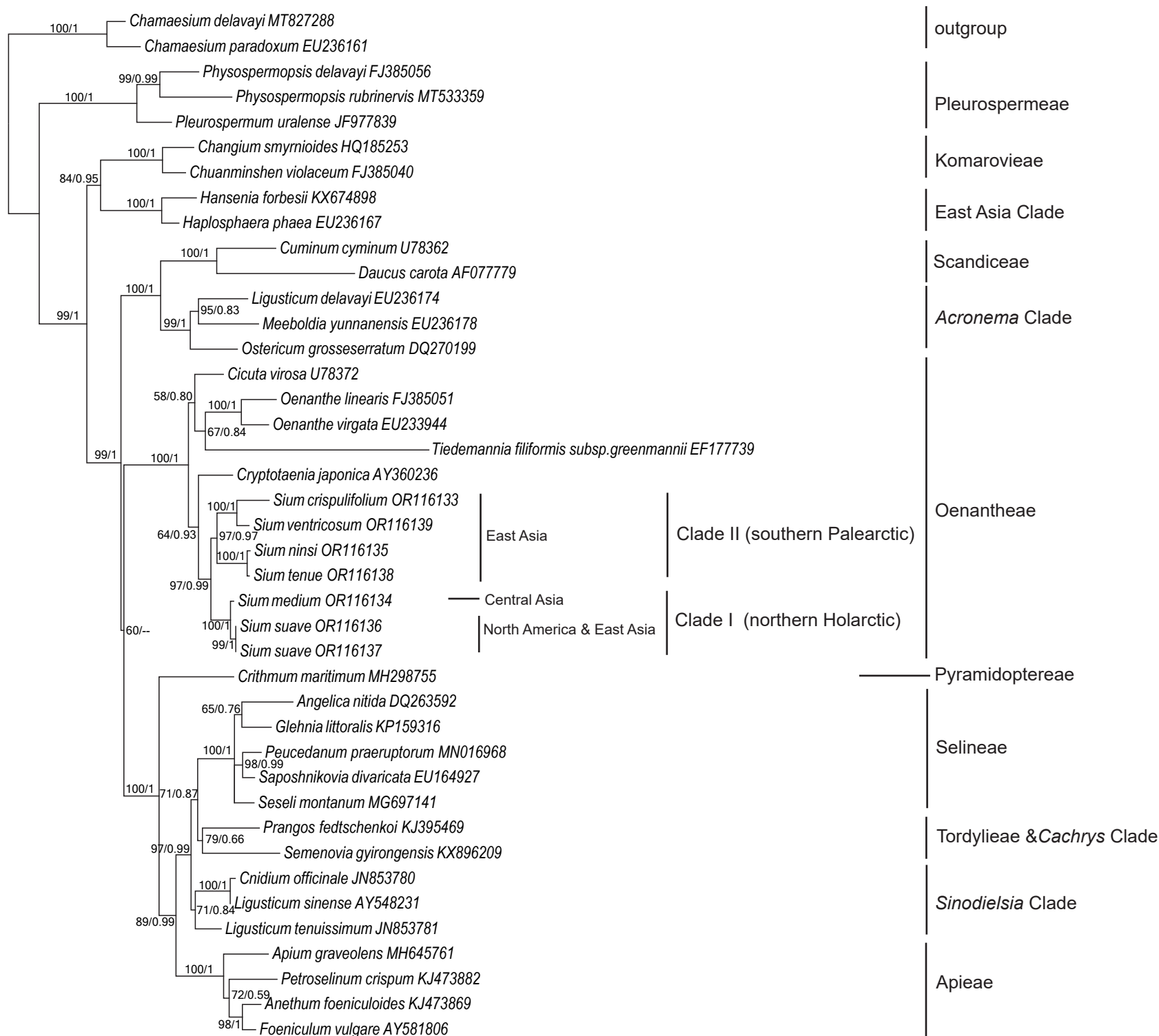

0.020

Supplement: Supplementary file 3 — Additional file 3: Figure S3. Phylogenetic relationships of Sium species inferred from Bayesian Inference (BI) and Maximum Likelihood (ML) analyses of the ITS sequences. Bootstrap values (BS) and posterior probabilities (PP) are shown next to the branches. – indicated that the branch has no support value from BI analysis. [file 12870_2023_4376_MOESM3_ESM.pdf]
