# Supplementary material for: Plastome evolution in the genus Sium (Apiaceae, Oenantheae) inferred from phylogenomic and comparative analyses
Source: BMC Plant Biol. 2023 Jul 25;23:368. doi: 10.1186/s12870-023-04376-8 (PMC10367252; doi:10.1186/s12870-023-04376-8)
Supplement: Supplementary file 4 — Additional file 4: Table S1. List of genes found in the Sium chloroplast genome. [file 12870_2023_4376_MOESM4_ESM.docx]

**Table S1**. List of genes found in the *Sium* chloroplast genome.

| **Category for genes** | **Group of genes** | **Name of genes** |
| --- | --- | --- |
| Photosynthesis | Rubisco | *rbcL* |
|  | Subunit of PhotosystemⅠ | *psaA*, *psaB*, *psaC*, *psaI*, *psaJ* |
|  | Subunit of PhotosystemⅡ | *psbA*, *psbB*, *psbC*, *psbD*, *psbE*, *psbF*, *psbH*, *psbI*, *psbJ*, *psbK*, *psbL*, *psbM*, *psbN*, *psbT*, *psbZ* |
|  | Subunit of ATP synthase | *atpA*, *atpB*, *atpE*, **atpF*, *atpH*, *atpI* |
|  | Subunit of cytochrome b/f compelx | *petA*, **petB*, **petD*, *petG*, *petL*, *petN* |
|  | Subunit of NADPH dehydrogenase | **ndhA*, **ndhB*(×2), *ndhC*, *ndhD*, *ndhE*, *ndhF*, *ndhG*, *ndhH*, *ndhI*, *ndhJ*, *ndhK* |
| Self-replication | Large subunit of ribosome | **rpl2*(×2), *rpl14*, **rpl16*, *rpl20*, *rpl22*, *rpl23*(×2), *rpl32*, *rpl33*, *rpl36* |
|  | DNA dependent RNA polymerase | *rpoA*, *rpoB*, **rpoC1*, *rpoC2* |
|  | Small subunit of ribosome | *rps2*, *rps3*, *rps4*, *rps7*(×2*)*, *rps8*, *rps11*, **rps12*(×2), *rps14*, *rps15*, **rps16*, *rps18*, *rps19* |
|  | tRNA genes | **trnA-UGC*(×2), *trnC-GCA*, *trnD-GUC*, *trnE-UUC*, *trnF-GAA*, *trnfM-CAU*, *trnG-GCC*, **trnG-UCC*, *trnH-GUG*, **trnI-GAU*(×2), *trnI-CAU*(×2), **trnK-UUU*, *trnL-CAA*(×2), **trnL-UAA*, *trnL-UAG*, *trnM-CAU*, *trnN-GUU*(×2), *trnP-UGG*, *trnQ-UUG*, *trnR-ACG*(×2), *trnR-UCU*, *trnS-GCU*, *trnS-GGA*, *trnS-UGA*, *trnT-GGU*, *trnT-UGU*, *trnV-GAC*(×2), **trnV-UAC*, *trnW-CCA*, *trnY-GUA* |
|  | rRNA genes | *rrn5*(×2), *rrn4.5*(×2), *rrn16*(×2), *rrn23*(×2) |
| Other genes | Subunit of Acetyl-CoA-carboxylase | *accD* |
|  | Maturase | *matK* |
|  | Translational initiation factor | *infA* |
|  | ATP-dependent protease | ***clpP* |
|  | c-type cytochrom synthesis gene | *ccsA* |
|  | Envelope membrane protein | *cemA* |
| Unknown function | Conserved open reading frames | *ycf1*, *ycf2* (×2), ***ycf3*, *ycf4*, *ycf15*(×2) |

Note: (×2): Two gene copies in IRs; *: gene containing a single intron; **: gene containing two introns.
