# Supplementary material for: Plastome evolution in the genus Sium (Apiaceae, Oenantheae) inferred from phylogenomic and comparative analyses
Source: BMC Plant Biol. 2023 Jul 25;23:368. doi: 10.1186/s12870-023-04376-8 (PMC10367252; doi:10.1186/s12870-023-04376-8)
Supplement: Supplementary file 6 — Additional file 6: Table S3. Primers for PCR amplification of the 12 variable regions among the seven Sium taxa. [file 12870_2023_4376_MOESM6_ESM.docx]

Table S3. Primers for PCR amplification of the 12 variable regions among the seven *Sium* taxa.

| No. | Region amplified | Forward primer (5’→3’) | Reverse primer (5’→3’) | Size (bp) | Annealing temperature (℃) |
| --- | --- | --- | --- | --- | --- |
| 1 | *trn*Q | ACTATCTACCTCACGACTCT | CGCCTAATCTGGACTTGTT | 1410 | 50 |
| 2 | *trn*G-*atp*A | TCTAATGGATAGGACAGAGGT | GCTATTCAGGAACAGATGGA | 1147 | 50.3 |
| 3 | *trn*E-*trn*T | AGCCACTAGCCACTATGA | GATGACTTACGCCTTACCA | 227 | 49.5 |
| 4 | *rps*4-*trn*T | TCATGGCATAGTTGGAAGTT | GAGGTTAGAGCATCGCATT | 900 | 50.3 |
| 5 | *acc*D-*psa*I | CAATGGCAATGGCTTCTT | TCTGCGTTATGATACAAGTC | 1162 | 48.6 |
| 6 | *rpl*16 | AGTGCCTGAAGAGCGTAT | GAACCAACCATCAACTATAACC | 1278 | 50.8 |
| 7 | *ycf*1-*ndh*F | ATAGTCTCCTTCTTCCTGATAC | ACCAATCCTTCCTTGTTCTT | 2236 | 50 |
| 8 | *ndh*F-*rpl*32 | TGATGATCCAAGACCATACA | CTTCCACTTCCAGTTCCTAT | 2476 | 49.3 |
| 9 | *rpl*32-*trn*L | CGACGATTGACTATGAATAGG | CGAACCGAGATGCTCTAG | 1495 | 49.7 |
| 10 | *ndh*E-*ndh*G | TCGACTCGTAATCAATCCAT | CGAACTCGGAATCCAAATC | 837 | 49.6 |
| 11 | *ycf*1a | AACGGCTTCCATTATAGGTA | GGCGTCGTAGAGAATTGA | 859 | 49.3 |
| 12 | *ycf*1b | GTTGATTGTAGTTCGCCATT | ACACCTCGGAGTTCTGAA | 1626 | 50 |
